# A surrogate method for comparison analysis of salivary concentrations of Xylitol-containing products

**DOI:** 10.1186/1472-6831-8-5

**Published:** 2008-02-11

**Authors:** Christine A Riedy, Peter Milgrom, Kiet A Ly, Marilynn Rothen, Gregory Mueller, Mary K Hagstrom, Ernie Tolentino, Lingmei Zhou, Marilyn C Roberts

**Affiliations:** 1 Northwest/Alaska Center to Reduce Oral Health Disparities and Department of Dental Public Health Sciences, School of Dentistry, University of Washington, 1959 NE Pacific St., Box 357475 Seattle, WA USA; 2 Regional Clinical Dental Research Center, School of Dentistry, University of Washington, 1959 NE Pacific St., Box 357480 Seattle, WA USA; 3 Department of Biobehavioral Nursing and Health Systems, School of Nursing, University of Washington, 1959 NE Pacific St., Box 357266 Seattle, WA USA; 4 Department of Environmental & Occupational Health Sciences, School of Public Health and Community Medicine, University of Washington, 1959 NE Pacific St., Box 357234, Seattle, WA USA

## Abstract

**Background:**

Xylitol chewing gum has been shown to reduce *Streptococcus mutans *levels and decay. Two studies examined the presence and time course of salivary xylitol concentrations delivered via xylitol-containing pellet gum and compared them to other xylitol-containing products.

**Methods:**

A within-subjects design was used for both studies. Study 1, adults (N = 15) received three xylitol-containing products (pellet gum (2.6 g), gummy bears (2.6 g), and commercially available stick gum (Koolerz, 3.0 g)); Study 2, a second group of adults (N = 15) received three xylitol-containing products (pellet gum, gummy bears, and a 33% xylitol syrup (2.67 g). For both studies subjects consumed one xylitol product per visit with a 7-day washout between each product. A standardized protocol was followed for each product visit. Product order was randomly determined at the initial visit. Saliva samples (0.5 mL to 1.0 mL) were collected at baseline and up to 10 time points (~16 min in length) after product consumption initiated. Concentration of xylitol in saliva samples was analyzed using high-performance liquid chromatography. Area under the curve (AUC) for determining the average xylitol concentration in saliva over the total sampling period was calculated for each product.

**Results:**

In both studies all three xylitol products (Study 1: pellet gum, gummy bears, and stick gum; Study 2: pellet gum, gummy bears, and syrup) had similar time curves with two xylitol concentration peaks during the sampling period. Study 1 had its highest mean peaks at the 4 min sampling point while Study 2 had its highest mean peaks between 13 to 16 minutes. Salivary xylitol levels returned to baseline at about 18 minutes for all forms tested. Additionally, for both studies the total AUC for the xylitol products were similar compared to the pellet gum (Study 1: pellet gum – 51.3 μg.min/mL, gummy bears – 59.6 μg.min/mL, and stick gum – 46.4 μg.min/mL; Study 2: pellet gum – 63.0 μg.min/mL, gummy bears – 55.9 μg.min/mL, and syrup – 59.0 μg.min/mL).

**Conclusion:**

The comparison method demonstrated high reliability and validity. In both studies other xylitol-containing products had time curves and mean xylitol concentration peaks similar to xylitol pellet gum suggesting this test may be a surrogate for longer studies comparing various products.

## Background

Xylitol, a naturally occurring sugar alcohol currently approved for use in foods, pharmaceuticals and oral health products in more than 35 countries has been shown to reduce cariogenic bacteria and tooth decay [1-5]; particularly delivered by either gum or lozenge [6]. Our previous studies [2,7] have reported on the minimally effective dose and frequency of use of xylitol delivered via pellet chewing gum to reduce *Streptococcus mutans*, a tooth decay pathogen. It is thought that xylitol's significant anti-caries effect is a result of constant S.mutans suppression [8] and alteration in virulence [9] from frequent and chronic exposure to xylitol-containing products. Although xylitol chewing gum is effective, new innovative ways to deliver xylitol at clinically effective levels are needed because chewing gum is neither safe, for the very young, nor acceptable, in schools, in the U.S. [6]. Nevertheless, repeated long-term clinical studies of the various xylitol vehicles are not feasible. Thus, the development of a surrogate test of a xylitol delivery system is warranted.

There have been two studies examining salivary xylitol levels after the intake of various xylitol-containing products in children [10,11], and none in adults. Lif Holgerson et al [10] determined salivary xylitol concentrations in children (mean age 11.5 years) after using a variety of xylitol-containing products (chewing gums, lozenges, candies, rinse, and fluoride toothpaste). All products were consumed in a single session with a brief rinse of distilled water immediately following consumption and a 10 min washout between xylitol products. It appears that saliva was collected from the floor of the mouth with a pipette. This resulted in recovering a large amount of the xylitol in the product but the method lacks any fidelity to actual conditions under which the oral flora is exposed to xylitol. Furthermore, it was unclear if saliva was collected the same way after consumption of the xylitol product. The researchers found that the xylitol products elevated salivary xylitol concentrations between eight to 16 minutes after xylitol product use. Tapiainen et al [11], in a second study, found salivary xylitol concentrations in preschool-aged children were immediately elevated after chewing gum or when a xylitol syrup was squirted into the mouth with a syringe but were undetectable after 15 minutes. Saliva was collected in a different manner depending on the age of the child; under age 3 saliva was collected directly from the mouth via a pipette while children age 4 and older spat through a funnel into test tubes. It is unclear how and when saliva was collected during xylitol administration in these children as the protocol is not detailed. For both studies, salivary xylitol concentrations were determined by enzymatic assay using a polyol dehydrogenase-based procedure (Boehringer Mannheim, Germany). The assay has a detection limit of approximately 200 ng/mL.

This paper describes a valid and reliable method to compare salivary xylitol concentrations during use of xylitol-containing products using a standardized and powerful method of chemical analysis, high performance liquid chromatography (HPLC). HPLC is a method of separating, identifying, and quantifying compounds in a sample. It has been used previously to measure xylitol and other sugar alcohols in gum and confectionary products [12,13]. For the HPLC assay, the lower limit of detection of xylitol, using the standard curve, is 0.2 ng/mL. Validity is the extent to which a test accurately measures the desired phenomenon it is attempting to measure [14]. Reliability is the extent to which the test is in agreement, where agreement may occur across two time periods (e.g., test-retest), between comparable forms of the same test, between individual sections of a test, or among different raters [15-17]. Two studies which address the presence and time course of xylitol in saliva delivered via a variety of xylitol-containing products are described. This paper specifically examines the presence and time course (peak and duration of detectable amounts) of xylitol concentrations in saliva for xylitol chewing gum (pellet and stick forms), xylitol gummy bear, and xylitol syrup; and compares the total xylitol-saliva time course curve (area under the curve) of xylitol pellet chewing gum to a commercially available xylitol stick gum, xylitol gummy bear and xylitol syrup.

## Methods

### Design

**Figure 1:**
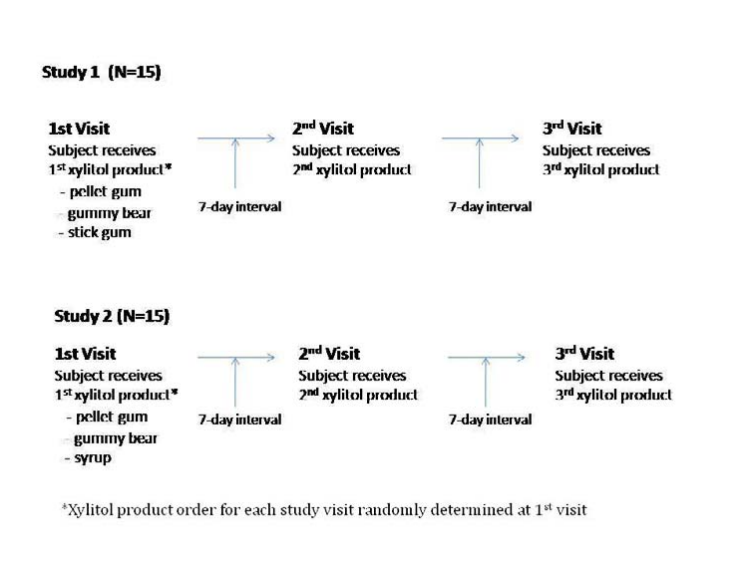
Within-subjects study design utilized for Studies 1 and 2.

Two studies were conducted using a within-subjects design for each study (See Figure 1).

### Subjects

For Studies 1 and 2, subjects (N = 15 per study) were recruited using advertising flyers and posters on the University of Washington, Seattle, WA campus. Potentially interested subjects were asked to call or email to learn more about the study. Subjects were screened using a list of questions (by phone) or a questionnaire (by email) to determine if they were eligible to participate. Subjects were excluded if they were under 18 years old, in poor health, had full or partial bridges, had implants, had phenylketonuria, or regularly used xylitol products. Xylitol use was determined by a food questionnaire that was developed and used for our previous xylitol studies and updated for these studies [2,7]. The food questionnaire was administered to potential subjects during the screening process. Regular xylitol use was defined as greater than 3 times a day or for greater than 3 days. These parameters were established because in our previous xylitol studies [2,7], this frequency was below the effective daily threshold. The Institutional Review Board of the University of Washington approved these studies and the written informed consent of the participants was obtained.

Eligible subjects were given a date and time for their initial (first) visit and asked to refrain from consuming any xylitol product 24 hours before their visit. Subjects received a list of products to avoid before their study visits. Subjects were also asked to refrain from eating or drinking (except water) 1 h prior to the visit.

### Procedures

#### General Procedures

At the initial (first) visit and subsequent visits, the above eligibility criteria were confirmed and a baseline stimulated saliva sample was collected. Subjects chewed paraffin for 1 minute (swallowed after the 1^st ^30 seconds, then allowed saliva to accumulate during the 2^nd ^30 seconds), spit into chilled pre-labelled 50 mL conical tubes, and discarded the paraffin. The tubes were placed on ice immediately after the collection. Subjects were eligible to continue in the study if they provided at least 1 mL of stimulated saliva at baseline of this first visit. After the baseline sample was taken, subjects received 1 of 3 xylitol-containing products. All subjects were tested with each of the 3 products over a 3-week period (See Studies 1 and 2). There was a 7-day washout between each product. The order of the xylitol products was randomly determined for each subject at this visit using a random number generator.

#### Study 1

For Study 1, after subjects provided a baseline saliva sample (described above), they consumed 1 of 3 xylitol products (xylitol pellet gum (2.56 g/visit in 3 pieces; Fennobon Oy, Karkkila, Finland), xylitol gummy bears (2.6 g/visit in 2 pieces; Santa Cruz Nutritional formerly Harmony Foods Corp, CA, U.S.), and xylitol stick gum (3.0 g/visit in 2 pieces; Koolerz, Hershey Foods, PA, U.S.) and gave saliva samples several times over a 15 to 20 minute period. Saliva samples were taken during stimulated (while using the xylitol product) and unstimulated (not using the product) time points. At each sampling time point, a minimum of 0.5 mL of saliva was collected.

For the xylitol pellet or xylitol stick gum visits, subjects chewed the gum and swallowed normally, at 1 minute they swallowed one last time, tilted their heads forward slightly, and spat the accumulated saliva into the pre-labelled 50 mL conical tube. This was repeated for sampling time points (2, 3, 4, and 5 minutes). After the 5 minute time point, the gum was discarded and subjects sat quietly with their heads hung, making minimal jaw and facial movements to allow the saliva to accumulate. The unstimulated saliva was collected at 7, 9, 11, 13, and 15 minutes post baseline.

For the gummy bear visit, subjects chewed and swallowed gummy bears (2 bears) one at a time. Immediately after consuming the first gummy bear, subjects swallowed one last time, tilted their heads forward slightly, and spat the accumulated saliva into the pre-labelled conical tube. Subjects followed the same procedure for consuming the second gummy bear. After the second gummy bear, subjects sat quietly with their heads hung, making minimal jaw and facial movements to allow the saliva to accumulate. The unstimulated saliva was collected at 4, 6, 8, 10, 12, 14, and 16 minutes post baseline.

#### Study 2

After subjects provided a baseline paraffin stimulated saliva sample in the same manner as in Study 1, they consumed 1 of 3 xylitol products (xylitol pellet gum (same dose as Study 1), xylitol gummy bears (same dose as Study 1), and 33% xylitol syrup (2.67 g/visit in a single 8 ml unit-dose applicator; formula available upon request) and gave saliva samples several times over a 15–20 minute period. As in Study 1 saliva samples were collected with a minimum of 0.5 mL per sample.

Refer to Study 1 for the procedures for the xylitol pellet gum and gummy bear visits. For the syrup visit, subjects applied approximately half of the syrup from the single use unit-dose applicator into each side of their mouths between their teeth and cheek. They swished the syrup around their teeth with their tongue before swallowing. Immediately after consuming the syrup, subjects swallowed one last time, tilted their head forward slightly, and spat the accumulated saliva into the pre-labelled conical tube. Subjects sat quietly with their heads hung, making minimal jaw and facial movements to allow the saliva to accumulate. Unstimulated saliva was collected at 2, 4, 6, 8, 10, 12, 14, 16, and 18 minutes.

For both Studies 1 and 2, saliva samples were transferred from the conical tubes to 1.5 mL Eppendorf tubes and frozen at -20°C prior to HPLC analysis.

### HPLC Analysis

High performance liquid chromatography (HPLC) was used to analyze the saliva samples. Saliva samples were treated as follows: 200 μL of the saliva sample was added to 50 μL of 1 M NaOH in a sterile 1.7 mL Eppendorf tube. To this, 50 μL of Mannitol (source) at 2 μg/mL was added to each sample to act as an internal standard. The samples were vortexed briefly and then pipetted into another tube fitted with a 0.45 micron PTFE filter and a clean autosampler vial. These samples were spun for 1 min at 2,000 rpm at room temperature; the eluant was used for HPLC analysis of monosaccharides on a CarboMA1 column [18 (Dionex Corp., Sunnyvale, CA)], and using the conditions listed below.

An ESA autosampler was used to inject 30 μL of sample during the separation run; the run time for each separation was 20 min. Isocratic HPLC separation of sugars was performed using degassed 500 mM NaOH as the eluant, utilizing a flow rate of 0.4 mL/min. The sugars were detected by a Pulsed Amperometric detector with the high-sensitivity Model 5040 gold electrodes (ESA Coulochem II electrochemical HPLC). In all cases, the pulse setting was +50, +700, and -800 mV for 500, 540, and 540 ms, respectively [19]. All run data was downloaded and analyzed using EZChrom software, version 2.0 (ESA, Chelmsford, MA). All salivary xylitol concentration data were expressed in μg/mL, but could be expressed in the Systeme International (SI) unit (mmol/L) (e.g., 40 μg/mL = 0.26 mmol/L).

### Data Analysis

All data were imported into MS Excel and checked. The mean (SD) xylitol concentration for each sampling point (Studies 1 and 2) was calculated using SPSS v. 12.02 (SPSS, Chicago, IL). Reliability was determined by calculating the correlation (Pearson product moment) between the xylitol chewing gum (pellet) and the other xylitol-containing products. For both studies approximation of area under the curve (AUC, μg.min/ml) for determining the average xylitol concentration in saliva over the total sampling period was calculated for each product using SAS/ETS PROC EXPAND (SAS, Cary, NC) and compared to the xylitol chewing gum (pellet) curve to determine validity of the method using PROC MIXED (SAS, Cary, NC).

## Results

### Study 1

**Figure 2:**
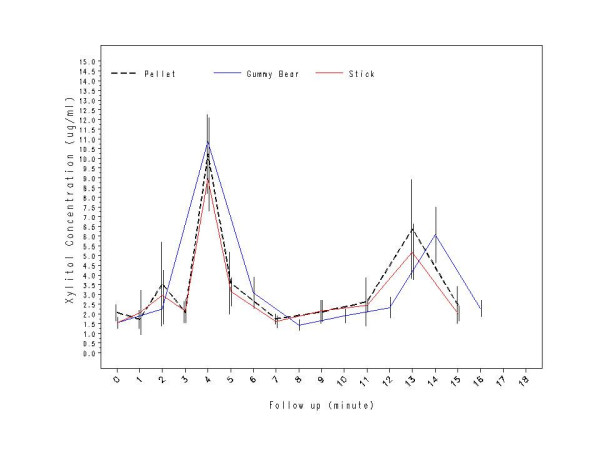
Study 1: Comparison of salivary xylitol concentrations (μg/mL) after using xylitol-containing products (N = 15).

In Study 1, all three xylitol products (pellet gum, gummy bears, and stick gum) had similar time curves with a higher first peak concentration followed by a lower second peak concentration during the sampling period (See Figure 2). Table 1 shows the mean xylitol concentrations for each xylitol product at designated sampling points. The mean first peak was at 4 min (pellet gum – 10.2 μg/mL; gummy bears – 10.9 μg/mL; and stick gum – 9.0 μg/mL). The mean second peak was at 13 min (pellet gum – 6.4 μg/mL; stick gum -5.2 μg/mL), and at 14 min (gummy bears – 5.7 μg/mL). The timing of the second peak between the xylitol gums and gummy bears can be attributed to the sampling protocol. Total AUC for the two xylitol-containing products did not differ significantly from the pellet gum (pellet gum – 51.3 μg.min/mL, gummy bears – 59.6 μg.min/mL, and stick gum – 46.4 μg.min/mL; F(1, 28) = 0.16, p = 0.69). The correlation coefficient (r^2^) between the mean xylitol concentrations at each time point was 0.99 between the xylitol pellet chewing gum and xylitol gummy bears and 0.99 between the pellet and stick gum.

**Table 1:** Study 1: Salivary xylitol concentrations ((μg/mL) over time; mean (SD)) after using xylitol-containing products (N = 15)

**Time**	**Pellet chewing gum 3 pieces × 0.85**	**Gummy bear 2 pieces × 1.3 g**	**Stick chewing gum 2 pieces × 1.5 g**
**Baseline**	2.06 (0.74)	1.55 (0.53)	1.55 (0.39)
**1 min**	1.72 (0.86)	1^st ^– 2.19 (1.11) 2^nd ^– 3.30 (1.58)	2.06 (2.08)
**2 min**	3.54 (3.89)	2.24 (1.39)	2.96 (2.28)
**3 min**	2.07 (0.98)	--------	2.16 (1.10)
**4 min**	10.22 (3.65)	10.87 (2.18)	8.97 (3.03)
**5 min**	3.59 (2.88)		3.14 (1.30)
**6 min**	------------	2.87 (1.56)	------------
**7 min**	1.75 (.47)	---------	1.60 (0.55)
**8 min**	------------	1.42 (0.49)	------------
**9 min**	2.10 (1.07)	---------	2.15 (0.99)
**10 min**	------------	1.90 (0.62)	------------
**11 min**	2.62 (2.25)	------------	2.43 (0.62)
**12 min**	------------	2.33 (0.96)	------------
**13 min**	6.37 (4.41)	------------	5.18 (2.60)
**14 min**	------------	5.66 (2.86)	------------
**15 min**	2.45 (1.65)	------------	2.03 (0.67)
**16 min**	------------	2.27 (0.76)	------------

### Study 2

**Figure 3:**
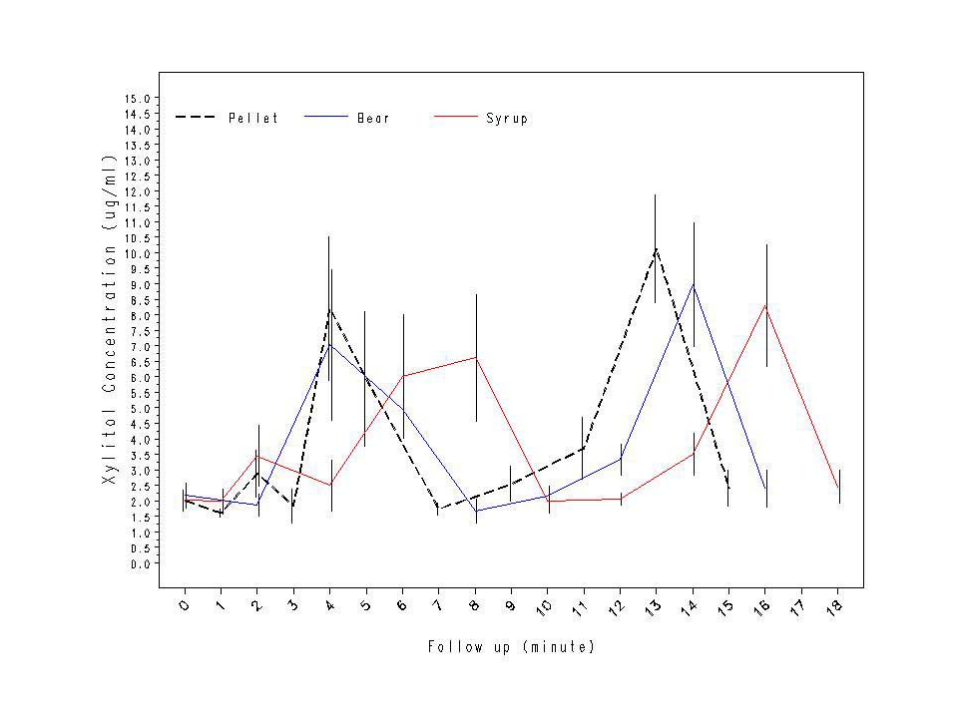
Study 2: Comparison of salivary xylitol concentrations (μg/mL) after using xylitol-containing products (N = 15).

In Study 2, the three xylitol products (pellet gum, gummy bears, and syrup) had similar time curves with a higher second peak concentration opposite to that of Study 1 (See Figure 3). Table 2 shows the mean xylitol concentrations at designated sampling points. The mean first peak was at 4 min (pellet gum – 8.2 μg/mL; gummy bears – 7.0 μg/mL) and at 6–8 min for syrup – 6.0 to 6.6 μg/mL. The mean second peak was between 13–16 min (13 min for pellet gum – 10.1 μg/mL; 14 min for gummy bears – 9.0 μg/mL; and 16 min for syrup -8.3 μg/mL). As in Study 1, the differential timing of the second peak between the xylitol products can be attributed to its different sampling protocols. Total AUC for the two xylitol-containing products did not differ significantly from the pellet gum (pellet gum – 63.0 μg.min/mL, gummy bears – 55.9 μg.min/mL, and syrup – 59.0 μg.min/mL; F(1, 26) = 0.05, p = 0.83). The correlation coefficient (r^2^) between the mean xylitol concentrations at each time point was 0.99 between the xylitol pellet chewing gum and gummy bears and was 0.96 between the pellet chewing gum and the syrup.

**Table 2:** Study 2: Salivary xylitol concentrations ((μg/mL) over time; mean (SD)) after using xylitol-containing products (N = 15)

**Time**	**Pellet chewing gum 3 pieces × 0.85**	**Gummy bear 2 pieces × 1.3 g**	**Syrup 2.67 g**
**Baseline**	2.02 (0.60)	2.19 (0.64)	2.01 (0.43)
**1 min**	1.61 (0.26)	1^st ^– 1.66 (0.48) 2^nd ^– 3.27 (0.96)	1.98 (0.73)
**2 min**	2.87 (1.35)	1.87 (0.60)	3.45 (1.76)
**3 min**	1.83 (1.00)	--------	------------
**4 min**	8.20 (4.20)	7.03 (4.00)	2.50 (1.47)
**5 min**	5.93 (3.91)		------------
**6 min**	------------	4.96 (1.49)	6.01 (3.62)
**7 min**	1.73 (0.33)	---------	------------
**8 min**	------------	1.66 (0.64)	6.61 (3.71)
**9 min**	2.54 (1.01)	---------	------------
**10 min**	------------	2.15 (0.56)	1.98 (0.68)
**11 min**	3.69 (1.81)	------------	------------
**12 min**	------------	3.33 (0.83)	2.06 (0.37)
**13 min**	10.12 (3.15)	------------	------------
**14 min**	------------	8.97 (3.28)	3.51 (1.21)
**15 min**	2.40 (1.05)	------------	------------
**16 min**	------------	2.40 (0.97)	8.29 (3.54)
**18 min**	------------	------------	2.45 (0.95)

## Discussion

The xylitol-containing products used in these studies were either commercially available (pellet gum, stick gum) or manufactured for another one of our studies (gummy bears, syrup). Subjects were instructed to consume the products as they normally would be consumed. For example, all gum pieces were chewed together and gummy bears were consumed one at a time.

Both studies showed that across all xylitol products xylitol salivary levels increased with xylitol product consumption. The xylitol amount (i.e., dose) consumed for each xylitol-containing product was the same unit dose previously determined in our xylitol studies to reduce *S. mutans *level when consumed 3 to 5 times per day [7].

Both studies showed that the other xylitol-containing products were similar to xylitol-containing pellet chewing gum ("the gold standard") in their level and time course of salivary xylitol concentrations. This suggests that this method is a valid way to measure xylitol concentration in saliva. Furthermore, the other xylitol-containing products' (gummy bears, xylitol stick gum, and xylitol syrup) mean salivary xylitol concentrations across time were highly correlated to xylitol pellet chewing gum's salivary xylitol. This suggests that this method is reliable.

Studies 1 and 2 concurred with the Lif Holgerson and Tapiainen studies [10,11]: salivary xylitol concentrations were elevated for a short period of time (less than 15 minutes) and then subsequently declined. Interestingly, the salivary levels within our studies did not increase in a linear fashion but in a bimodal fashion. Both the Lif Holgerson and Tapiainen studies [10,11] did not show a second peak concentration. As previously mentioned, it is unclear from the description of the Lif Holgerson and Tapiainen studies of the specific protocols followed for saliva sampling. Our study protocol was geared toward a "real life" approach; subjects were instructed to consume the xylitol products as they normally would. It is possible that the two peaks demonstrated in the current studies were a result of the protocol; i.e., sampling both stimulated (during xylitol consumption) and unstimulated salivary levels since all products sampled demonstrated this. In the Lif Holgerson study (Experiment A), saliva was collected after consumption of the xylitol product (unstimulated saliva) which would correspond with our unstimulated sampling period, and from the floor of the mouth using a pipette. This may be an artefact of the salivary collection system and raises questions about the validity of this approach. Additionally, in the Tapiainen study only one sample per child was taken whereas in the current study repeated samples were taken per subject. Finally, the limits of detection between the current and cited studies were different. The HPLC analysis had a much lower level of detection of xylitol (0.2 ng/mL) compared to the enzymatic assay (0.2 mg/L) used in the previous studies [10,11].

Interestingly, Study 2 had xylitol salivary levels slightly below Study 1 and had its highest mean peak concentration at the latter half of the sampling period compared to Study 1. It is unclear why Study 2 results showed the reverse bimodal distribution of salivary xylitol levels. This variation may have been due to the inherent differences of another group of subjects used in Study 2.

## Conclusion

This methodology can be useful for examining the salivary concentration and time course of xylitol in other xylitol-containing products and could serve as a surrogate for longer and more expensive studies involving bacterial level assessment or clinical trials involving a large number of human subjects. For both studies, compared to the xylitol pellet gum, the other xylitol-containing products tested had similar time curves and mean xylitol concentration peaks. This suggests that the other xylitol products' effects on bacterial levels and tooth decay should be similar to the effects demonstrated with xylitol pellet gum use.

## Competing interests

The author(s) declare that they have no competing interests.

## Authors' contributions

CAR participated in the design and analysis of the study and drafted the manuscript. PM conceived of the study, participated in the design and analysis, and commented on the manuscript. KAL participated in the design and analysis of the study and commented on the manuscript. MR, GM, MKH, MCR carried out the study, and ET conducted the HPLC assay, and commented on the manuscript. LZ assisted in the data analysis. All authors read and approved the final manuscript.

## Pre-publication history

The pre-publication history for this paper can be accessed here:


